# Prognostic value of precise hepatic pedicle dissection in anatomical resection for patients with hepatocellular carcinoma

**DOI:** 10.1097/MD.0000000000019475

**Published:** 2020-03-06

**Authors:** Hui Zhao, Wen-Zhou Ding, Hong Wang, Shen Gu, Xiao-Peng Yan, Shi-Quan Sun, Liang Mao, Hui-Han Jin, Yu-Dong Qiu

**Affiliations:** a Department of Hepatopancreatobiliary Surgery, the Affiliated Wuxi No. 2 People's Hospital of Nanjing Medical University, Wuxi; b Department of Hepatopancreatobiliary Surgery, Drum Tower Hospital, Medical School of Nanjing University; c Hepatobiliary Center, The First Affiliated Hospital of Nanjing Medical University, Nanjing, Jiangsu, China.

**Keywords:** anatomical resection, hepatocellular carcinoma, precise hepatic pedicle dissection, propensity score matching analysis

## Abstract

The present study aimed to investigate the long-term and perioperative outcomes of precise hepatic pedicle dissection in anatomical resection (precise AR) vs non-anatomical resection (NAR) for hepatocellular carcinoma (HCC) patients.

Data from a total of 270 consecutive HCC patients who underwent curative hepatectomy were retrospectively collected. Propensity score matching (PSM) analysis was performed. The long-term outcomes of precise AR and NAR were analyzed using the Kaplan–Meier method and the Cox proportional hazards model.

The 1-, 3-, and 5-year overall survival (OS) rates were 90.3%, 76.2%, and 65.7% in the PS-precise AR group, respectively (n = 103); and 88.3%, 70.5%, and 52.0% in the PS-NAR group, respectively (n = 103) (*P* = .043). The 1-, 3-, and 5-year recurrence-free survival (RFS) rates were 83.4%, 63.2%, and 46.0% in the PS-precise AR group, respectively; and 75.7%, 47.4%, and 28.3% in the PS-NAR group, respectively (*P* = .002). Multivariate analysis showed that ICG-R15, BCLC staging, and microvascular invasion (MVI) were independent risk factors for OS; while tumor size, types of resection, surgical margin, and MVI were independent risk factors for RFS. Subgroup analysis indicated that the RFS rate was significantly better in the PS-precise AR group than in the PS-NAR group for patients with MVI and tumor size ≤5 cm.

After PSM, precise hepatic pedicle dissection in AR significantly improved the recurrence-free survival rate of solitary HCC patients compared with NAR, especially in those with MVI and tumor size ≤5 cm.

## Abstract 2

Supplemental Digital Content is available in the text

## 1 Introduction

Hepatocellular carcinoma (HCC) is the sixth most frequent cancer and the third most common cause of cancer-related mortality in the world.^[1,2]^ It has been reported that more than 60% of the HCC patients are complicated with liver cirrhosis.^[3]^ In East Asia, especially in mainland China, hepatitis B virus (HBV) infection is the most common cause of liver cirrhosis.^[4]^ Liver resection and transplantation are considered as the first-line surgical treatment of HCC with cirrhosis.^[5]^ Unfortunately, due to the serious shortage of organ donors and high medical expense, liver transplantation is rarely performed in China. Therefore, hepatectomy is still the first choice therapy for early-stage HCC patients after precise preoperative evaluation.

Research works have shown that micrometastases caused by the spread of HCC cells through the portal venous system result in intrahepatic recurrence.^[6,7]^ Anatomical resection (AR), compared to non-anatomical resection (NAR), has been proved to have the advantage of a lower rate of recurrence.^[8]^ Theoretically, AR can completely remove the tumor-bearing portal tributaries and eradicate possible intrahepatic micrometastases to improve the prognosis of HCC.^[9–12]^ However, there is no standard procedure for AR. From 2010, we developed the technique for precise hepatic pedicle dissection in AR based on the concept of precise liver resection. This technique mainly includes preoperative 3D reconstruction of liver vessels, a dual surgical approach through hepatic portal and hepatic veins, a precise clamp crushing method, and intermittent portal triad occlusion for liver parenchymal transection.

In the present research, we retrospectively investigated the long-term outcomes of precise hepatic pedicle dissection in AR compared with NAR in solitary HCC patients, using a propensity score matching (PSM) analysis method to eliminate possible selection bias.

## 2 Methods

### 2.1 Patients

The study cohort consisted of 351 consecutive HCC patients who underwent curative hepatectomy between January 2010 and December 2015 at the Affiliated Wuxi No. 2 People's Hospital of Nanjing Medical University. HCC patients with multiple tumors, recurrent tumors, macroscopic vascular invasion, R1 resection, Child-Pugh B/C, and any preoperative anticancer treatments were excluded. The remaining 270 patients were included in this research. Patients were then divided into the following two groups according to the different hepatectomy methods used: precise hepatic pedicle dissection in the AR group (precise AR group) and the NAR group. All operations were carried out by the same group of surgeons. This research was conducted in accordance with the *Declaration of Helsinki*, and it was approved by the Committee of Medical Ethics of the Affiliated Wuxi No. 2 People's Hospital of Nanjing Medical University. The work has been reported in line with the STROCSS criteria.^[13]^

### 2.2 Perioperative factors

Preoperative factors included patient age, sex, cirrhosis, hepatitis B virus surface antigen (HBsAg), serum alanine aminotransferase (ALT), serum aspartate aminotransferase (AST), gamma glutamyl transpeptidase (GGT), alkaline phosphatase (AKP), serum total bilirubin (TB), direct bilirubin (DB), serum albumin (ALB), alpha-fetoprotein (AFP), platelet count (PLT), international normalized ratio (INR), Child-Pugh classification, ICG-R15, MELD score, BCLC staging, tumor size, surgical time, surgical margin, blood loss, and blood transfusion. All surgical specimens were examined macroscopically and microscopically to determine surgical margins. Microvascular invasion (MVI) was defined as microscopic tumor invasion in a portal vein or hepatic vein of the surrounding hepatic tissue.^[14]^

### 2.3 Surgical procedure

#### 2.3.1 The precise AR group

The hepatic pedicle of the liver segment planned for resection was located by CT vessels 3D reconstruction before the operation and intraoperative ultrasound. Hepatic vein branches running between segments were also identified.

**Figure 1:**
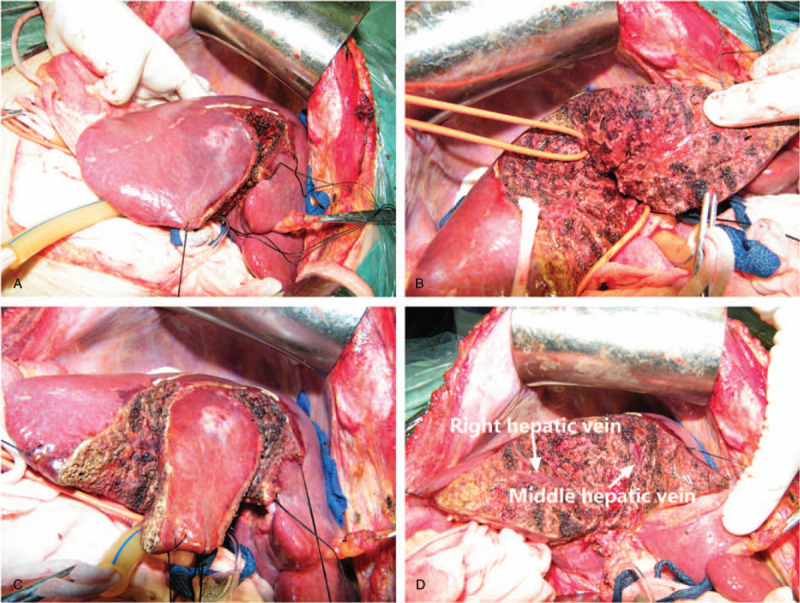
The precise hepatic pedicle dissection in S5 anatomical segmentectomy for a patient with hepatocellular carcinoma.

Right hepatic segmentectomy or sectoriectomy (Fig. 1): Right and middle hepatic arteries, together with right anterior and posterior branches of the portal vein were freed at the first hepatic hilum. Then the corresponding right anterior or posterior branches of the portal vein were blocked according to the required resection range. After clarifying the ischemia range, intraoperative ultrasound was used to locate the hepatic vein or the branches between hepatic segments as the marker of liver parenchymal transection. The located hepatic vein branches running between segments were found 2 cm deep into the liver parenchyma, and they provided the direction to perform liver parenchymal transection. We took advantage of the hand-cross formed by the hepatic vein and Glisson system to ferret out the hepatic pedicle of the resected segments, and tried to perform dissection and transection after confirming the entire focus range. All hepatic pedicle branches running to the hepatic segments with tumors were cut in the same way, and then relative hepatic segmentectomy or sectoriectomy was performed.

Left hepatic segmentectomy or sectoriectomy: Dissection of the outer segment was performed in the sagittal section of the hepatic portal vein. Following that, the hepatic pedicle branches of the resected segments were consecutively ligated and cut.

#### 2.3.2 The NAR group

NAR was defined as local resection or enucleation irrespective of the Couinaud segmental, sectoral structure.

### 2.4 Patient follow-up

After the operation, patients were routinely followed up by performing the serum AFP and imaging examinations, such as ultrasonography and computed tomography (CT), every 3 months. Overall survival (OS) was described as the time interval between the operation and death. Recurrence-free survival (RFS) was described as the time interval between the operation and the date of the first recurrence. Follow-up data were collected until December 31, 2017

### 2.5 Statistical analysis

The PSM analysis was used to reduce the influence of selection bias and potential confounders generated by the imbalance of perioperative factors between precise AR and NAR. Variables with potential influence on the outcomes, such as age, gender, cirrhosis, HBsAg, MELD score, ICG-R15, BCLC staging, ALT, AST, TB, DB, AKP, GGT, Albumin, INR, Platelet, AFP, tumor size, MVI, blood loss, transfusion, tumor differentiation, and surgical margin, were included in PSM. The propensity score, which was calculated by using the logistic regression model, predicted the probability of a patient receiving a proper operation procedure. PSM was accomplished by using the 1:1 nearest-neighbor matching method within a caliper width of 0.10. Perioperative factors after PSM were compared using the Wilcoxon signed rank test for continuous variables and the McNemar test for categorical variables.^[15]^ PSM was performed using SAS version 9.2 (SAS Institute Inc., Cary, NC). Continuous variables, presented as the median and range, were analyzed by the Mann–Whitney *U* test. Categorical data were analyzed by the Chi-square test and Fisher exact test. The survival analysis was performed using the Kaplan–Meier survival curves. The log-rank test was used to compared OS and RFS. The Cox proportional hazards models were used to identify the risk factors for OS and RFS. The stratified log-rank test and the stratified Cox proportional hazard models for the propensity score were performed in survival, univariate, and multivariate analyses.^[15]^ A two-tailed *P* < .05 was considered to be statistically significant. Statistical analysis was performed using SPSS version 21.0 (SPSS Inc., Chicago, IL).

## 3 Results

### 3.1 Patients’ baseline and surgical characteristics before and after PSM

Out of the 270 HCC patients, 136 patients (50.4%) received precise AR, whereas 134 patients (49.6%) received NAR. This cohort included 216 males and 54 females. As presented in Tables 1 and 2, no significant differences in age, gender, cirrhosis, HBsAg, ALT, albumin, platelets, AFP, tumor size, BCLC staging, MVI, blood loss, and transfusion were observed between the precise AR and NAR groups. However, liver function variables such as MELD score, ICG-R15, AST, TB, DB, GGT, AKP, and INR were significantly better in the precise AR group than those in the NAR group. In terms of the surgical characteristics, surgical margin and operation time were significantly increased in the precise AR group. After the 1:1 PSM, 103 patients were included in each group (PS-precise AR group and PS-NAR group). Between the 2 groups, no significant differences were observed in each variable except for the operation time. In terms of the surgical outcomes, no significant difference was observed in total length of hospital stay, operative mortality, and postoperative complications between the 2 groups before and after PSM.

**Table 1:**
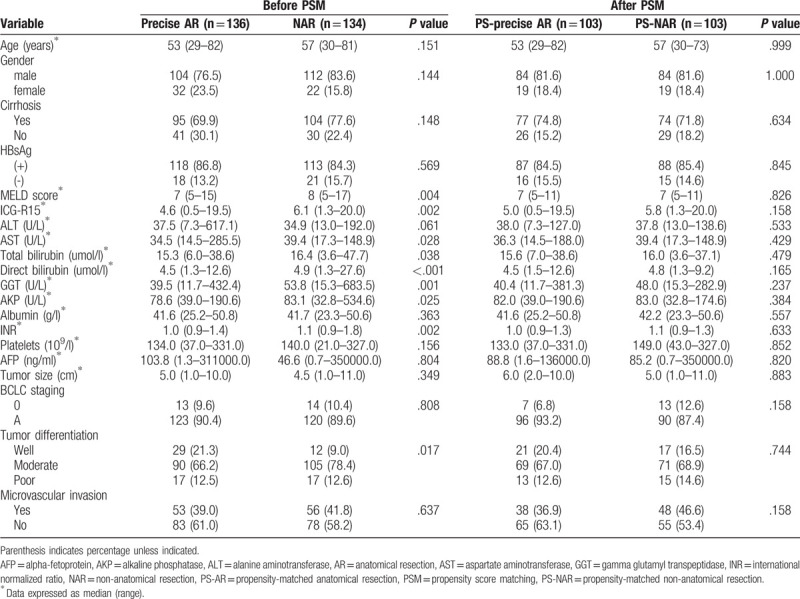
Baseline characteristics of the precise AR group and NAR group before and after PSM.

**Table 2:**
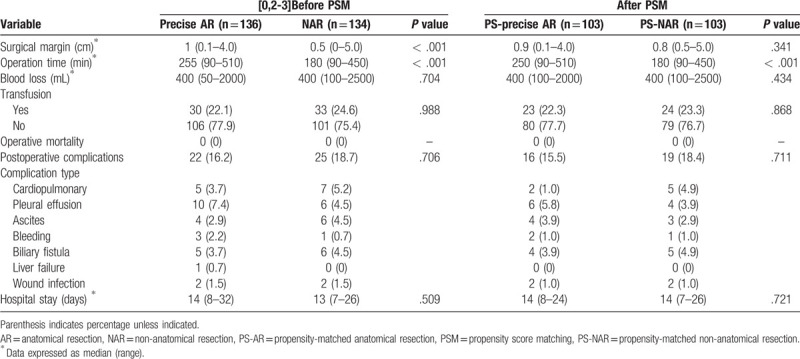
Surgical characteristics and outcomes of the precise AR group and NAR group before and after PSM.

### 3.2 Overall survival analysis of all patients before and after PSM

**Figure 2:**
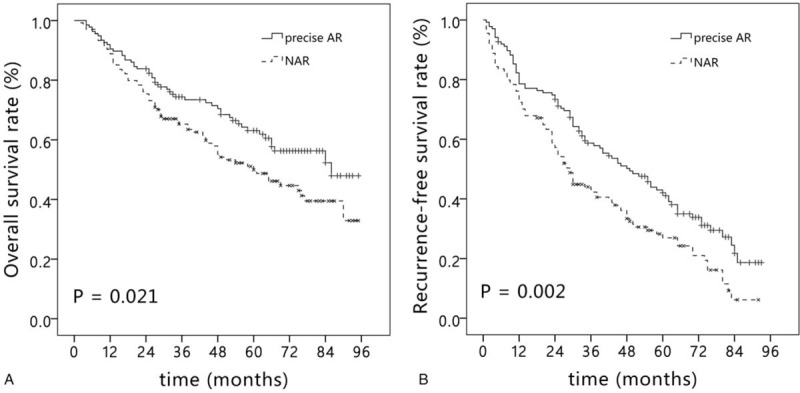
Kaplan–Meier curve of overall survival (A) and recurrence-free survival (B) in the precise AR group and NAR group before propensity score matching. AR = anatomical resection, NAR = non-anatomical resection.

**Figure 3:**
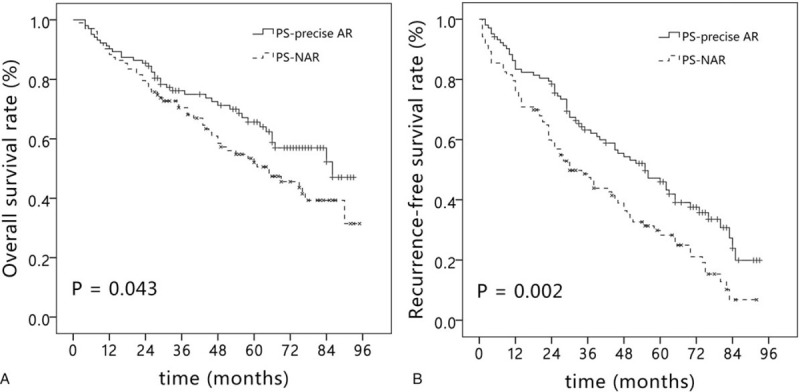
Kaplan–Meier curve of overall survival (A) and recurrence-free survival (B) in the precise AR group and NAR group after propensity score matching. AR = anatomical resection, NAR = non-anatomical resection.

The mean follow-up time after the operation was 52 months (range, 2–95 months). The 1-, 3-, and 5-year OS rates were 90.4%, 74.4%, and 61.9%, respectively, in the precise AR group; and 88.8%, 65.3%, and 49.9%, respectively, in the NAR group (*P* = .021) (Fig. 2A). After PSM, the 1-, 3-, and 5-year OS rates were 90.3%, 76.2%, and 65.7%, respectively, in the PS-precise AR group; and 88.3%, 70.5%, and 52.0%, respectively, in the PS-NAR group (*P* = .043) (Fig. 3A). Univariate analysis showed that ICG-R15, BCLC staging, tumor size, types of resection, tumor differentiation, surgical margin, and MVI were associated with the OS rate (Supplementary Table 1). In multivariate analysis, ICG-R15, BCLC staging, and MVI were identified as independent risk factors for the OS rate (Table 3).

**Table 3:**
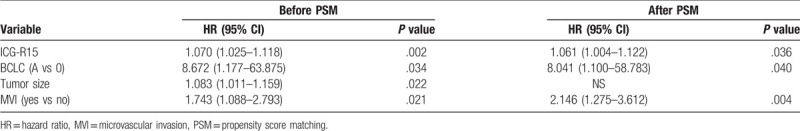
Multivariate analysis of prognostic factors of overall survival before and after propensity score matching.

### 3.3 Recurrence-free survival analysis of all patients before and after PSM

The 1-, 3-, and 5-year RFS rates were 78.6%, 58.7% and 42.1% in the precise AR group, respectively; and 72.4%, 43.2%, and 27.0% in the NAR group, respectively (*P* = .002) (Fig. 2B). After PSM, the 1-, 3-, and 5-year RFS rates were 83.4%, 63.2%, and 46.0% in the PS-precise AR group, respectively; and 75.7%, 47.4%, and 28.3% in the PS-NAR group, respectively (*P* = .002) (Fig. 3B). Tumor size, blood loss, types of resection, tumor differentiation, surgical margin, and MVI affected the RFS rate in the univariate analysis (Supplementary Table 2). The Cox multivariate analysis identified tumor size, types of resection, surgical margin, and MVI as independent risk factors for the RFS rate (Table 4).

**Table 4:**
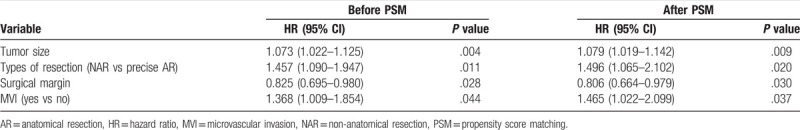
Multivariate analysis of prognostic factors of recurrence-free survival before and after PSM.

### 3.4 Subgroup analysis after PSM according to MVI and tumor size

**Figure 4:**
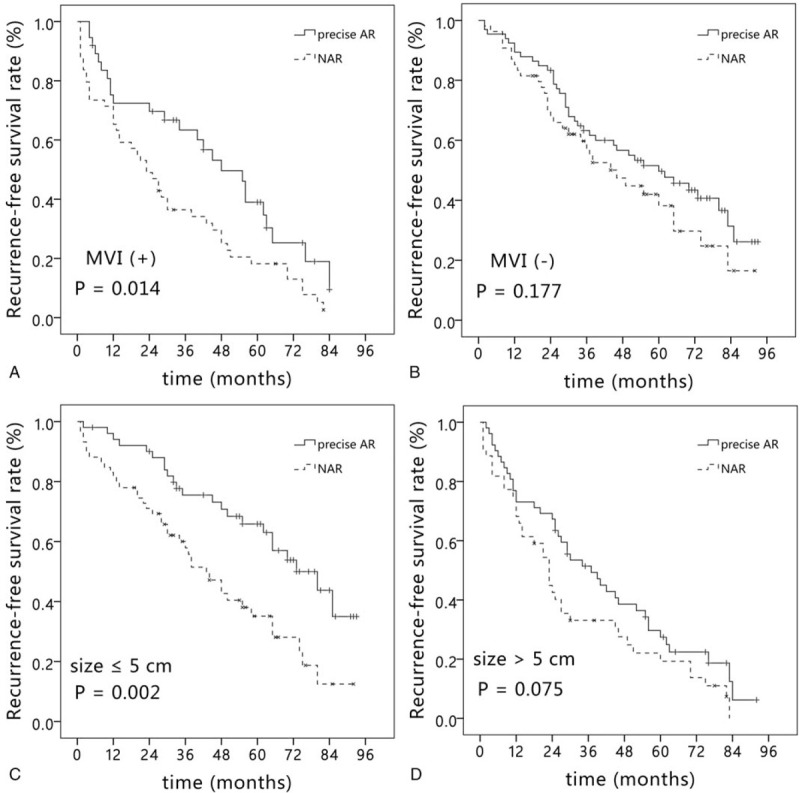
Kaplan–Meier curve of recurrence-free survival in the precise AR group and NAR group with and without MVI (A, B), tumor size ≤ 5 cm and > 5 cm. (C, D). AR = anatomical resection, MVI = microvascular invasion, NAR = non-anatomical resection.

To evaluate the influence of MVI and tumor size on the results of precise AR and NAR, subgroup analysis after PSM was performed in all HCC patients. In patients with MVI, precise AR significantly improve the RFS rate compared with NAR (*P* = .014, Fig. 4A). In patients without MVI, there was no significant difference in the RFS rate between the two groups (*P* = .177, Fig. 4B). In patients with tumor size ≤5 cm, precise AR provided better RFS rate compared with NAR (*P* = .002, Fig. 4C). While in patients with tumor size > 5 cm, no significant difference was observed in the RFS rate between the two groups (*P* = .075, Fig. 4D).

## 4 Discussion

In this retrospective study, we investigated the prognostic superiority of precise AR in solitary HCC patients. Precise AR significantly improved the RFS rate in patients compared with NAR, especially in patients with MVI and tumor size ≤ 5 cm. Liver resection is widely considered as one of the most valuable treatments for HCC.^[16]^ Unfortunately, the prognosis of liver resection is unsatisfactory because of the high recurrence rate.^[17]^ The most important cause of the high recurrence rate is the intrahepatic metastasis of HCC through the portal venous system. AR, which is defined as complete removal of the tumor-bearing portal tributaries, may prevent the development of intrahepatic metastases from HCC. Many previous research works suggested that AR provided better long-term outcomes compared with NAR.^[12,18,19]^ A meta-regression analysis by Cucchetti et al showed that the OS and RFS rates after AR were superior to those after NAR.^[20]^ However, Shindoh et al suggested that AR significantly decreased local tumor recurrence and improved disease-specific survival of HCC patients compared with NAR.^[12]^

However, AR is always associated with major hepatic resection, which might induce high incidence of postoperative liver failure, especially in HCC patients with cirrhosis. Therefore, the benefit of AR was questioned in some research works.^[21–23]^ There are 2 important reasons for this occurrence. First, in clinical practice, there has been a tendency to perform AR in HCC patients with good liver function and NAR in patients with poor liver function. Therefore, patient selection bias in these case-controlled studies reduced the strength of the results. In the present research, we only included solitary HCC patients with Child-Pugh class A, but significant differences were still observed in ICG-R15, MELD score, AST, TB, DB, GGT, AKP, INR, tumor differentiation, and surgical margin between the 2 groups. The liver function of HCC patients in the NAR group was obviously poorer than that in patients in the precise-AR group. Therefore, we tried to eliminate the selection bias and achieve a balance between the 2 groups by performing PSM, which was defined as the conditional probability of receiving a treatment given the value of covariates, and it can be used to adjust for the selection bias between the 2 groups.^[24]^

Second, there is no standard procedure for AR, especially for anatomical hepatic segmentectomy. It is important to confirm the borders of the liver segment on the liver surface and the liver parenchymal transection plane. Glisson system of the liver comprises the portal vein, the hepatic artery, and different branches of hepatic ducts wrapped in connective tissues. Each liver segment with an independent Glisson system is considered as an independent anatomic structure and functional unit. Takasaki^[25]^ designed the Glissonean pedicle transection method for liver resection. It is easy to ligate the hepatic pedicle of the resected segment in the hilar area and to guide liver resection according to the ischemic boundary of the liver. However, to block the corresponding hepatic pedicle in the hilar plate, it is necessary to dissect more fibrous connective tissues of the hilar plate and the gallbladder bed. For example, part of segment V needs to be resected to unveil the hepatic pedicle trunk of segment VI/VII and segment V/VIII. This approach might increase not only the risk of injury to the hepatic portal area and intrahepatic ducts but also the bleeding volume and operation time, which could lead to increase in the incidence of postoperative complications and mortality rate.

Due to the existence of communicating branches between segments, the ischemia line after hepatic pedicle amputation did not completely tally with the actual boundary between segments. Therefore, we improved the operation skill. The new approach could precisely dissect the hepatic pedicle of the required resected segment, and there was no need to excessively dissect more fibrous connective tissues of the hilar plate and the gallbladder bed. It is well known that the route of metastasis from liver cancer is fundamentally through the portal vein. During our surgical procedure, we mainly used the portal vein as the marker of the hepatic pedicle. When the indicated hepatic pedicle of the resected segment was identified, early ligation and occlusion was performed to cut the portal vein and its branches. When combined with the Pringle method (15-minute occlusion and 5-minute reperfusion) during the operation, this technique decreased the metastasis from the liver tumor and the postoperative recurrence rate. The RFS rate in the precise AR group was significantly better than that in the NAR group through PSM and the same surgical procedure in our research. During the surgical procedure, we always preferred to dissect through the liver parenchyma along the hepatic vein and its tributaries as this method could identify the inter-segmental demarcation plane. Additionally, since the backflow in the hepatic vein was prevented as much as possible, the functional structure of the remnant liver was maintained, which efficiently prevented the development of ischemia or necrosis at the edge of the remnant liver after surgery.

It has been reported that tumor size is an important factor in determining the type of resection. Eguchi et al divided 5781 HCC patients from Japan into three groups according to the tumor size (< 2 cm, 2–5 cm, and > 5 cm) ^[18]^. The benefit of AR with respect to the RFS rate was only observed in patients with a tumor size of 2 to 5 cm. However, another study from Japan showed that AR was not superior to NAR in terms of survival outcomes in patients with tumor size < 5 cm.^[22]^ In our study, we divided all patients into the following 2 groups: tumor size ≤5 cm and tumor size > 5 cm because of a low proportion of HCC patients with tumor size < 2 cm. The precise AR group provided better OS and RFS rates compared with the NAR group in patients with tumor size ≤ 5 cm, but no significant difference was found in patients with tumor size > 5 cm. It seems reasonable that the prognosis of large tumors might be affected by not only the type of resection but also the tumor characteristics. Additionally, anatomical hemihepatectomy and extended hemihepatectomy are always performed in patients with large tumors, which might increase the incidence of postoperative liver failure and offset the advantage of AR.

MVI was an independent prognosis risk factor for the OS and RFS rates in this study. Especially in patients with MVI, precise AR significantly improved the RFS rate compared with NAR. HCC has a high tendency to invade the intrahepatic vascular system and spreads through the branch, which is the main route for the formation of MVI. Precise AR could completely remove the tumor-bearing portal tributaries in order to eliminate the microscopic metastases in the liver.

The present research has several limitations. First, because of its retrospective design and nonrandomized nature, the possibility of selection bias cannot be ignored. However, we tried to eliminate the selection bias and achieve a balance between the 2 groups by performing PSM, which provided strong evidence for our results. Second, it was a single center study, and the sample size was relatively small. Therefore, larger multicenter studies should be performed to validate our results.

In conclusion, through preoperative assessment of hepatic functional reserve, a dual surgical approach through hepatic portal and hepatic veins, the clamp crushing method, and intermittent portal triad occlusion for liver parenchymal transection, precise hepatic pedicle dissection can achieve the goal of clearing the target focus thoroughly. The safety and feasibility of the surgical procedure were proved in our research. After PSM, precise hepatic pedicle dissection in AR significantly improved the prognosis of solitary HCC patients compared with NAR, especially in patients with tumor size ≤5 cm.

## Acknowledgments

We thank the whole multiple disciplinary team (MDT) in hepatobiliary cancer for their guidance in this study.

## Author contributions

**Conceptualization:** Hui Zhao, Hui-Han Jin.

**Data curation:** Wen-Zhou Ding, Shen Gu.

**Investigation:** Hong Wang, Xiao-Peng Yan.

**Methodology:** Shi-Quan Sun.

**Project administration:** Liang Mao.

**Visualization:** Wen-Zhou Ding.

**Writing – original draft:** Hui Zhao.

**Writing – review & editing:** Hui-Han Jin, Yu-Dong Qiu.

## Supplementary Material

Supplemental Digital Content
