# Supplementary material for: Prognostic value of precise hepatic pedicle dissection in anatomical resection for patients with hepatocellular carcinoma
Source: Medicine (Baltimore). 2020 Mar 6;99(10):e19475. doi: 10.1097/MD.0000000000019475 (PMC7478661; doi:10.1097/MD.0000000000019475)
Supplement: Supplemental Digital Content [file medi-99-e19475-s001.docx]

**Supplementary Table 1. Univariate analysis of prognostic factors of overall survival before and after propensity score matching**

|  | Before PSM | |  | After PSM | |
| --- | --- | --- | --- | --- | --- |
| Variable | HR (95% CI) | *P*-value |  | HR (95% CI) | *P*-value |
| Age | 0.990 (0.974-1.005) | 0.200 |  | 0.988 (0.970-1.006) | 0.194 |
| Gender  (male vs female) | 1.047 (0.831-1.318) | 0.698 |  | 1.318 (0.746-2.329) | 0.342 |
| MELD score | 1.045 (0.955-1.143) | 0.339 |  | 1.034 (0.884-1.209) | 0.677 |
| ICG-R15 | 1.079 (1.034-1.126) | < 0.001 |  | 1.065 (1.009-1.124) | 0.023 |
| BCLC (A vs 0) | 5.706 (1.814-17.954) | 0.003 |  | 6.494 (1.598-26.380) | 0.009 |
| ALT | 0.998 (0.994-1.002) | 0.308 |  | 0.998 (0.993-1.004) | 0.563 |
| AST | 1.001 (0.997-1.004) | 0748 |  | 1.002 (0.987-1.006) | 0.426 |
| Total bilirubin | 1.013 (0.996-1.030) | 0.129 |  | 1.010 (0.974-1.034) | 0.403 |
| Direct bilirubin | 1.038 (0.989-1.090) | 0.132 |  | 1.040 (0.965-1.120) | 0.303 |
| AKP | 1.002 (0.998-1.005) | 0.332 |  | 1.004 (0.997-1.011) | 0.264 |
| GGT | 1.002 (1.000-1.004) | 0.071 |  | 1.002 (1.000-1.005) | 0.095 |
| Albumin | 0.963 (0.923-1.006) | 0.090 |  | 0.972 (0.921-1.025) | 0.296 |
| INR | 2.398 (0.525-10.956) | 0.259 |  | 2.759 (0.290-26.227) | 0.377 |
| Platelet | 1.000 (0.998-1.003) | 0.780 |  | 1.000 (0.997-1.004) | 0.882 |
| AFP | 1.000 (1.000-1.000) | 0.013 |  | 1.000 (1.000-1.000) | 0.158 |
| Tumor size | 1.160 (1.101-1.221) | < 0.001 |  | 1.158 (1.089-1.232) | 0.011 |
| Operation time | 1.002 (1.000-1.004) | 0.075 |  | 1.001 (0.999-1.003) | 0.244 |
| Blood loss | 1.000 (1.000-1.001) | 0.050 |  | 1.000 (1.000-1.001) | 0.126 |
| Types of resection  (NAR vs precise AR) | 1.496(1.045-2.142) | 0.021 |  | 1.472 (1.026-2.226) | 0.044 |
| Tumor differentiation  (moderate/poor vs well) | 1.784 (1.039-3.063) | 0.036 |  | 2.056 (1.033-4.092) | 0.040 |
| Surgical margin | 0.729 (0.575-0.924) | 0.009 |  | 0.712 (0.544-0.932) | 0.013 |
| MVI (yes vs no) | 2.372 (1.656-3.397) | < 0.001 |  | 2.594 (1.706-3.944) | < 0.001 |
| Transfusion (yes vs no) | 0.865 (0.709-1.055) | 0.153 |  | 0.872 (0.692-1.098) | 0.243 |
| PSM = propensity score matching, ALT = alanine aminotransferase, AST = aspartate aminotransferase, GGT = gamma glutamyl transpeptidase, AKP = alkaline phosphatase, INR = international normalized ratio, AFP = alpha-fetoprotein, MVI = microvascular invasion. | | | | | |

**Supplementary Table 2. Univariate analysis of prognostic factors of recurrence-free survival before and after propensity score matching**

|  | Before PSM | |  | After PSM | |
| --- | --- | --- | --- | --- | --- |
| Variable | HR (95% CI) | *P*-value |  | HR (95% CI) | *P*-value |
| Age | 0.996 (0.983-1.009) | 0.515 |  | 0.992 (0.977-1.006) | 0.264 |
| Gender (male vs female) | 0.946 (0.665-1.344) | 0.755 |  | 1.186 (0.765-1.840) | 0.446 |
| MELD score | 0.969 (0.894-1.052) | 0.454 |  | 0.942 (0.827-1.074) | 0.376 |
| ICG-R15 | 1.024 (0.917-1.092) | 0.124 |  | 1.038 (0.990-1.087) | 0.120 |
| BCLC (A vs 0) | 1.726 (1.001-2.974) | 0.049 |  | 0.516 (0.262-1.013) | 0.055 |
| ALT | 1.001 (0.997-1.003) | 0.464 |  | 0.999 (0.996-1.003) | 0.756 |
| AST | 1.002 (1.000-1.005) | 0.073 |  | 1.001 (0.997-1.005) | 0.654 |
| Total bilirubin | 0.998 (0.982-1.015) | 0.841 |  | 0.990 (0.967-1.013) | 0.371 |
| Direct bilirubin | 1.009 (0.967-1.053) | 0.678 |  | 0.984 (0.921-1.052) | 0.636 |
| AKP | 1.001 (0.999-1.004) | 0.311 |  | 1.162 (0.841-3.096) | 0.132 |
| GGT | 1.001 (0.999-1.003) | 0.216 |  | 1.002 (0.999-1.004) | 0.150 |
| Albumin | 0.966 (0.932-1.000) | 0.053 |  | 0.969 (0.927-1.013) | 0.161 |
| INR | 1.084 (0.286-4.115) | 0.905 |  | 1.912 (0.278-13.142) | 0.510 |
| Platelet | 1.002 (1.000-1.004) | 0.126 |  | 1.002 (0.999-1.005) | 0.139 |
| AFP | 1.000 (1.000-1.000) | 0.051 |  | 1.000 (1.000-1.000) | 0.304 |
| Tumor size | 1.098 (1.051-1.148) | < 0.001 |  | 1.115 (1.058-1.175) | < 0.001 |
| Operation time | 1.002 (0.998-1.003) | 0.128 |  | 1.002 (0.999-1.004) | 0.134 |
| Blood loss | 1.000 (1.000-1.001) | 0.024 |  | 1.000 (1.000-1.001) | 0.041 |
| Types of resection  (NAR vs precise AR) | 1.585 (1.192-2.108) | 0.002 |  | 1.692 (1.214-2.357) | 0.003 |
| Tumor differentiation  (moderate/poor vs well) | 1.707 (1.128-2.583) | 0.011 |  | 2.074 (1.213-3.544) | 0.008 |
| Surgical margin | 0.784 (0.658-0.935) | 0.007 |  | 0.758 (0.621-0.927) | 0.007 |
| MVI (yes vs no) | 1.579 (1.189-2.098) | 0.002 |  | 1.669 (1.201-2.321) | 0.002 |
| Transfusion (yes vs no) | 0.960 (0.816-1.129) | 0.619 |  | 0.964 (0.796-1.168) | 0.707 |
| PSM = propensity score matching, ALT = alanine aminotransferase, AST = aspartate aminotransferase, GGT = gamma glutamyl transpeptidase, AKP = alkaline phosphatase, INR = international normalized ratio, AFP = alpha-fetoprotein, MVI = microvascular invasion. | | | | | |
